# Green synthesis of silver nanoparticles, graphene, and silver-graphene nanocomposite using *Melissa officinalis* ethanolic extract: Anticancer effect on MCF-7 cell line

**DOI:** 10.22038/IJBMS.2022.65503.14410

**Published:** 2023-01

**Authors:** Farzaneh Motafeghi, Mahyar Gerami, Parham Mortazavi, Babak Khayambashi, Nasrin Ghassemi-Barghi, Mohammad Shokrzadeh

**Affiliations:** 1 Department of Pharmacology and Toxicology, Faculty of Pharmacy, Mazandaran University of Medical Sciences, Sari, Iran; 2 Ministry of Science, Research and Technology, Department of Plant Physiology Biology, Sana Higher Education Institute, Mazandaran, Sari, Iran; 3 Student Research Committee, Faculty of Pharmacy, Mazandaran University of Medical Sciences, Sari, Iran; 4 Soil and Water Research Department, Isfahan Agricultural and Natural Resources Research and Education Center, AREEO, Isfahan, Iran; 5 Toxicology and Diseases Group (TDG), Pharmaceutical Sciences Research Center (PSRC), The Institute of Pharmaceutical Sciences (TIPS), Tehran University of Medical Sciences, Tehran, Iran

**Keywords:** Cell death, Comet assay, DNA damage, Melissa, MCF-7 cells, Nanoparticles, Oxidative stress, Silver graphene

## Abstract

**Objective(s):**

Nanotechnology has helped a lot in diagnosing and treating multiple illnesses, specifically cancer, and increasing the development of targeted drug delivery methods. Nanocomposites are materials with at least one component smaller than 100 nm. Therefore, this study aims to assess the anticancer effects of silver-graphene nanocomposite on MCF-7.

**Materials and Methods:**

In this study, the rate of inhibition of cancer cell growth and production of reactive oxygen radicals, malondialdehyde, and glutathione stores in MCF7 cells were investigated. Cancer cells were exposed to nano particles for 48 hr. Silver nanoparticles and graphene both reduced the growth rate of MCF-7.

**Results:**

Subsequently, by treating the cells with silver-graphene nanocomposite, the rate of inhibition of cell growth at the highest concentration was 84.60%. Nanoparticles also inhibited the growth of cancer cells through the oxidative stress pathway by increasing the amount of intracellular ROS, followed by increasing malondialdehyde and decreasing glutathione stores, so that at the highest combined concentration of nanoparticles, the amounts of LPO and ROS increased up to 70% and 74 %, and glutathione reserves decreased by 16%.

**Conclusion:**

Treatment of MCF-7 cells with silver or graphene nanoparticles and combination treatment with these two substances against cisplatin have sound effects, and by affecting oxidative stress factors, such as increased ROS and subsequent increase in lipid membrane damage, inhibit cell growth and proliferation. According to the mathematical model, silver graphene nanocomposite> silver nanoparticles> graphene has the best effect in inhibiting the growth of cancer cells, respectively.

## Introduction

In today’s world, nanotechnology has greatly helped to diagnose and treat illnesses, specifically cancer, by using nanoparticles and developing new targeted drug delivery methods (1, 2). The most important of nanoparticle effects is the one on cancer cells. In progressing and developing new methods to administer cancer drugs, the main goal is to reach malignant tissues without poisoning the entire body. The size of nanoparticles gives them unique properties to enter and exit tumors (3). Nanoparticles smaller than 10 nm are quickly excreted by the kidneys, and nanoparticles bigger than 100 nm have difficulty getting through tumors. Nanoparticles between 10 and 100 nm can enter blood circulation to find the tumor. However, they cannot enter healthy cells through the capillary wall, whereas tumors’ capillary wall is abnormal and contains large holes for the nanoparticles to enter. Nanoparticles tend to gather inside tumors while having minimal effects on other body parts (4). Nanoparticles enter cells using endocytosis and are far from the reach of cellular pumps (5). Studies have shown that low concentrations of nanomaterials can destroy cancer cells (6).

Nanoparticles can be divided into various types. According to the morphology, physical, size, and chemical properties. Some are carbon-based nanoparticles, ceramic nanoparticles, metal nanoparticles, polymeric nanoparticles, semiconductor nanoparticles, and lipid-based nanoparticles (7). Besides, nanoparticle types are commonly divided into two main groups: organic and inorganic. The first group consists of micelles, liposomes, dendrimers, hybrid, and compact polymeric nanoparticles. The second group contains quantum dots, fullerenes, and metal and silica nanoparticles (8, 9).

Cancer cells use the mechanism of autophagy to cope with stress and survive. As stress increases, autophagy is activated, destroying ROS and stopping the aging mechanism (10, 11). The results of preclinical studies have shown that, by inhibiting autophagy, anticancer drugs are more effective. This mechanism also creates resistance to CDK4 / 6 inhibitors. Inhibition of CDK4 / 6 itself inhibits autophagy and cancer growth. As intracellular ROS increases, AKT, an oncogenic factor, is activated (12, 13).

*Melissa officinalis* L. (MO) is a plant of the *Lamiaceae* family. This plant has different names, such as lemongrass, bee mummy, and honey mummy, and in Iran, it is known as Badranjbouyeh, Warangboo, and Faranjmashk in some places. The growing regions of this plant are in southern Europe and northern Africa, the Caucasus and northern Iran, the eastern Mediterranean region, and western Asia. Ingredients of this plant include rosmarinic acid, chlorogenic acid, caffeic acids, tannins, flavonoids, and sesquiterpenes (11, 14).

Studies have shown that *Melissa officinalis* has antiproliferative and apoptotic effects on cancer cells. One of the functional mechanisms of this plant is ROS formation and induction of apoptosis (15, 16). Numerous studies on different cell lines have shown that inhibiting the growth of cancer cells is dose-independent, indicating that the optimal biological dose is more important than the maximum tolerable dose. In addition, the antiproliferative effects appear to be different for each type of tumor because hormone-dependent cancers are more sensitive to the antitumor effects of this plant (14, 17).

Consumption of this plant in prescribed doses, topically or orally, or in food for up to 30 days in healthy adults is not prohibited, and no side effects have been reported for this plant. Generally, this plant in the United States is classified as safe (GRAS) with a maximum level of 0.5% in cooked products (18).

One of the methods of making nanoparticles is biosynthesis. In this method, natural materials such as plants and microorganisms are used to synthesize nanoparticles. Plants are a viable option due to their compatibility with the body and environment. The biosynthesis of nanoparticles using plant extracts has its perks in pharmacology and other medical applications and is considered a simple replacement for complicated physical and chemical methods. In this technique, extracts from biological organisms are used both as stabilizers and reducers in the production of nanoparticles. In this nanoparticle production process, in contrast to physical and chemical methods that have 2 phases and require an oxidation-reduction phase and a phase of adding a stabilizer, biological methods are single-phase, and the oxidizer and stabilizers are already in biological extracts. In conclusion, this process is energy efficient (19).

Amongst nanostructures, metallic nanoparticles are the ones with biological applications, where 56% of nanoparticles happen to be silver ones (20). The properties of silver nanoparticles can be described as quick, non-toxic, hypoallergenic, able to withstand different conditions, hydrophilic, compatible with the environment, heat resistant, and effective on bacteria, fungi, and viruses (21). The anticancer properties of silver have been proven in studies. Silver can disrupt the cellular communication network but fault cell signaling(22, 23). Also, silver nanoparticles can cause DNA damage, increase the expression of molecular protein caspase 3, and start cellular apoptosis. The ability to cause apoptosis makes them essential in cancer treatment (24).

Graphene is one of the carbons’ allotropes. It has a honeycomb structure consisting of single plates of carbon atoms. Its unique qualities, including lightness and thinness, electrical, optical, thermal properties, and mechanical resistance, make it useful in multiple fields such as electrical, thermal, sensitizers, refining industry, textile industry, capacitor production, and as medicine carriers for tissue engineering in pharmacology (25). The binding of the drug to graphene through the non-covalent interaction of aromatic drug molecules and the role of a large area can be used as an example. Since in graphenes’ structure, every carbon atom is connected to 3 other atoms, every atom has a singular free electron that can overlap to make the Pi orbital and causes pi with pi interaction. This quality is most used in drug delivery systems for controlled drug loading and release (26). 

Therefore, this research aims to study the anticancer effect of *M. officinalis* coated with silver, graphene, and silver-graphene nanocomposite on breast cancer cell line by evaluating oxidative stress factors.

## Materials and Methods

Materials

In this study, silver-graphene nanocomposite and silver nanoparticles were synthesized using the Salunke and Kim method (27). The MCF-7 cell line breast cancer (ATCC Number: HTB-22) was purchased from the Pasteur Institute of Iran. All reagents used in the tests were purchased from Sigma Company in a laboratory grade.

***Preparing the extracts of melissa officinalis***

The ethanolic extraction method was chosen to produce an extract from *M. officinalis* leaves (ethyl alcohol and water 1:1). Five grams of *M. officinalis leaves* were put into 25 ml of the ethanolic solvent. They were pounded for 15 min, and then 90 ml of extra solvent was combined with the mashed content. The extraction was done using a mechanical stirrer for 3 hr with a 750 rpm rotational speed in a dark area. The extracted solution was filtered twice using Whatman Grade 1 filter papers. The extracts were placed in a dark tube at 4 °C (28). 

***Preparing the metal nanoparticles (AgNP) suspension***

Ten ml of plant extract was combined with 1 mmol/laqueous AgNo3 solution (90 ml) to reduce the Ag+. Afterward, the solution at 60 °C was stirred for 2 hr. It was centrifuged for 20 min at 15000 rpm to purify the synthesized nanoparticles with pellet re-dispersion in the deionized water. The purified powder of nanoparticles was used to aid us in further analyses (28). 

***Synthesizing graphene***

In order to materialize graphene, plant extract (50 g/l) was placed in the low-power sonication of expanded graphite (Samsung C & G, Korea, 1-10 g/l) for 24 hr. The temperature was lowered to less than 30 °C and stabilized there using a continuous flow of water in the ultra-sonication bath (JACUltra-sonic4020P). In order to separate large unstable graphite aggregates, the dispersion was left overnight after the sonication process. Water with the sodium cholate surfactant at 0.3 mg/ml was used to steady graphene dispersions. 46 ml of deionized water was added to the paste, and the solution was evaporated at 95 °C for 40 min. The solution was centrifuged at 1500 rpm for 90 min to obtain a stable dispersed graphene solution. The final solution was washed with distilled water to remove the sulfate ions. The solution was dried at 50 °C for two days using an air oven. The final powder was used for the subsequent analysis (28).

***Preparing nanocomposites suspension***

Graphene and silver ions were reduced together so that the Ag/Graphene nanocomposites could be prepared. Firstly, 1 g of graphite was combined with 100 ml of AgNP solution and mixed with 50 ml of deionized water. The reaction mixture was sonicated for one day in the ultra-sonication bath (JAC ultrasonic 4020P) at 60 °C. 10 ml of hydrazine using magnetic stirring was slowly added to the mixture. The sonication temperature was organized by controlling the water volume in the bath and covering it with a lid. Then for one hour, the mixture’s temperature was raised to 95 °C and kept at that level. The gained powders were washed numerous times using ethanol and deionized water. After centrifugation, an air oven was used to dry the prepared samples at 50 °C for 48 hr (28).

***Characterization of nanocomposites***

The amount of UV-visible and measurable nanocomposites was determined using a UV-visible spectrophotometer (UV-1601, Shimadzu, Japan). The nanocomposite solution was frozen, and its structure and composition were analyzed using field emission SEM (Carl Zeiss, LEO-1530). The X-ray differentiation method was used (Bruker AXS, D8 Discover with GADDS) to analyze AgNP and graphene. A Nano Raman spectroscope (NT-MDT, NTEGRA) was used to discuss the structural properties of graphene sheets (single-layer or multilayer properties) (28). The particle size, the size distribution of the nanoparticles (PDI), and zeta potential were assessed using a particle size analyzer (zeta analyzer, Malvern, UK). 

Preparation of MCF7 cell line

The MCF-7 cell line was positioned in the culture medium containing 1% AB (penicillin/streptomycin) and 10% FBS (fetal bovine serum). The culture medium containing the cells was incubated at 37 °C and 5% CO_2_. In order to execute the tests, when the cells reached 70% of cellular growth, they were separated from the bottom of the flask using Trypsin-EDTA, and the contents were centrifuged for 5 min at 1500 rpm. A suspension was created using 1 ml of the cell culture and the sedimented cells. The percentage of live cells in the cell suspension was determined using a trypan blue dye, a hemocytometer slide, and a light microscope. After ensuring that the cells in the suspension were not contaminated, cells with a higher than 90% survival rate were used for the test (29, 30).

***Assessing cell toxicity using the MTT test***

In order to determine cell toxicity, the breast cancer cells (from the MCF-7 line) were exposed to different concentrations of the assembled mixtures. It is a competitive metabolic test and evaluates mitochondrial function. The yellow-colored MMT, which happens to be a tetrazole, is reduced to the insoluble purple-colored formazan in the mitochondria of live cells. The reduction only happens in the presence of active mitochondrial reductase enzymes. Therefore, this reaction can be directly related to the number of live cells. Afterward, different concentrations of the suggested combination (silver nanoparticles and silver-graphene nanocomposite) were prepared. The negative control group received no stimulant, the positive control group was exposed to an IC50 dose of cisplatin (5.75 µM)(31), and the treatment group was exposed to different concentrations of silver-graphene nanocomposites and silver nanoparticles (15, 30 ,60, 120, and 250 μg/ml) based on pre-tests optimum results (30).

Calculating the amount of ROS

The amount of ROS was determined using fluorometry and the DCFH-DA reagent in the control and treatment groups. 20 μl of DCFH-DA was added to the cell sample and kept at the temperature of 4 °C for 15 min. Absorption was then measured at the wavelength of 312 nm and the emission of 420 nm (30).

Measurement of lipid peroxidation

After incubation, the cells were assessed using the thiobarbituric acid (TBA) method. To 0.2 ml of cell suspension, 0.1 ml of TBA reagent was added. The mentioned materials were mixed well, placed in a hot water bath, and incubated for 30 min. After cooling down, 0.2 ml of n-butanol was added to the combination, shaken well, and centrifuged for 10 min at 3500 rpm. The n-butanol layer was separated at the wavelength of 532 nm, and the amount of TBARs was calculated using the standard curve (30). 

***Determining the amount of in-cell glutathione***

In the tubes containing cells, 1.5 ml of TCA was added. The samples were centrifuged for 15 x 3500 g min to precipitate the proteins. Then, 2.5 ml of Tris buffer and 0.5 ml of DTNB were added to the supernatant solution and incubated for 15 min. The tube was shaken well until a uniform yellow color appeared. Finally, absorbance was measured spectrophotometrically at 412 nm (32).

***Comet assay***

First, 1% NMP (normal melting agarose) was used to cover the slides. Then, 1 ml of LMP 1% (low melting agarose) was added to the treated cells, and slides coated with 1% NMP agarose were placed in this solution so that a layer was placed on them and kept at 4 °C for ten min. The slides were immersed in an alkaline lysis buffer and placed in an alkaline electrophoretic buffer for 40 min to decompress the DNA. Then, electrophoresis was performed for 40 min (300 mA and 25 V). The slides were immersed in neutralizing Tris buffer solution for 15 min and stained with ethidium bromide for ten min. A fluorescence microscope with 400x magnification was used to examine the slides. DNA damage was measured using Comet score software and expressed as tail length, and percentage of DNA in tail and tail moment, and the results were presented as mean ± SEM (33).

Statistical analysis

The statistical program Prism ver. 6 was used to analyze data. Statistical tests, ANOVA and Tukey’s post test were used for further, more complete analyses. The amount of IC_50_ was determined using the Sigma plot program.

Mathematical models were analyzed by SPSS Software, and graphs were made by software Table Care 2D ver. 5.01.

## Results

***SEM images of synthesized AgNP / graphene nanocomposite on M. officinalis leaves by SEM images***

**Figure 1:**
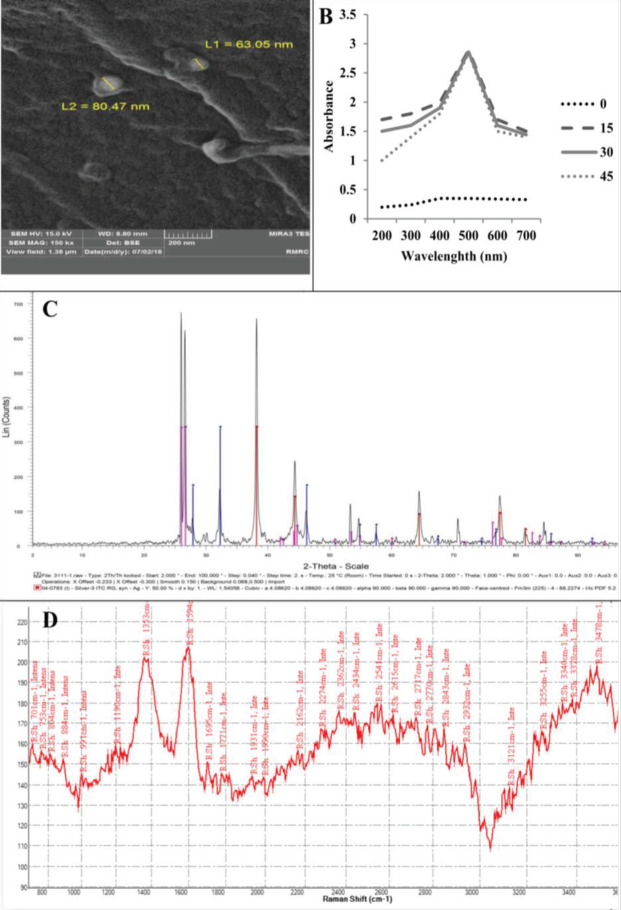
Characterization of AgNP and graphene synthesized with *Melissa officinalis* L., size, and shape

Synthetic silver nanoparticles are shown. AgNP has a diameter of about 50 to 80 nm and a spherical shape (Figure 1a). In UV-vis spectra, the peak is 480 nm for M. officinalis extract (300 to 700 nm) (Figure 1b). The results of XRD data indicate that AgNP was successfully prepared from M. officinalis plant extract (Figure 1c). Raman spectroscopy has been used to detect the crystal structure of graphene, which (Figure 1d) confirms the crystal structure of graphene. For plant extract samples, two peaks at about 1353 and 1594 cm−1 were assigned to the D and G-band. The D-band and G-band are associated with the vibrations of sp3 and sp2 carbon atoms of disordered graphene nanosheets. It was recognized that the G and 2D bands of the peak showed monolayer graphene sheets (28).

Table 1 shows the particle size, zeta potential, and size distribution (polydispersity index (PDI) of particles measured by Zetasizer (Malvern Instruments, Worcestershire, UK), based on the dynamic light scattering (DLS) technique.

***Assessing the antiproliferative effect of nanoparticles on MCF-7 cell line***

***According to*** Table 2, the treatment group at 15 μg/ml concentration reduced the cell proliferation by 38,6%, 25.60%, and 45.60%, respectively. With increasing concentration, we saw a decrease in growth so that, at 250 μg/ml, the rate of proliferation of MCF7 was reduced to 77.2%, 68.4%, and 86.40%, respectively. Also, cisplatin, which was used at IC_50_ concentration to treat cancer cells, had a growth inhibition rate of 52.20%.

**Figure 2:**
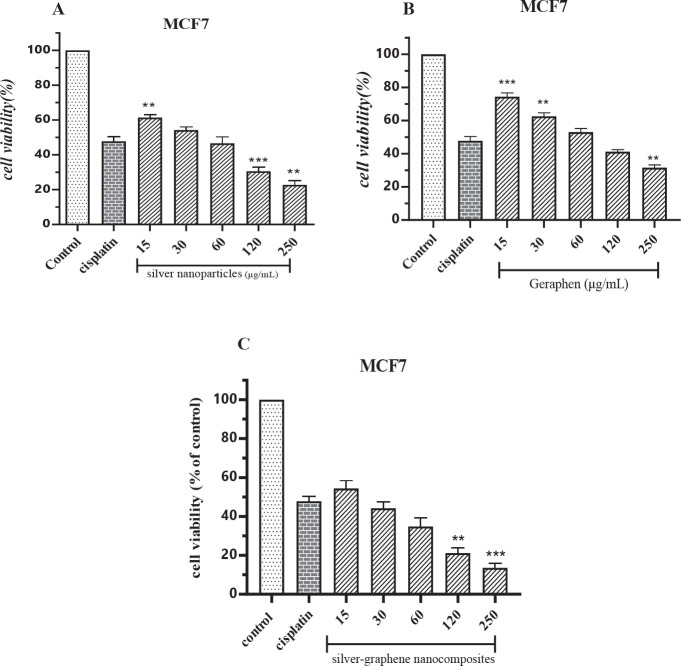
A. Comparing the effects of different concentrations of silver nanoparticles on the toxicity levels of the MCF-7 breast cancer cell line using the MTT method. ** (*P*<0.01), ***(*P*<0.001) compared to cisplatin group. B. Comparing the effects of different concentrations of graphene on the toxicity levels of MCF-7 breast cancer cell line using the MTT method. ** (*P*<0.01), ***(*P*<0.001) compared to cisplatin group. C. Comparing the effects of different concentrations of silver-graphene nanocomposites on the toxicity levels of MCF-7 breast cancer cell line using the MTT method. ** (*P*<0.01), ***(*P*<0.001) compared to cisplatin group

Compared with the cisplatin group, the silver nanoparticles group, at concentrations of 15 μg/ ml (*P*<0.01), 120 μg/ ml (*P*<0.001), and 250 μg/ml (*P*<0.01) were significantly different (Figure 2A). Concentrations of 15 μg/ ml (*P*<0.001), 30 μg/ ml (*P*<0.01), and 250 μg/ mL(*P*<0.01) of the graphene group were significantly different (Figure 2B). Silver-graphene nanocomposites group had significant differences only in concentrations of 120 and 250 μg/ ml (*P*<0.01 and *P*<0.001, respectively) (Figure 2C).

***Amount of reactive oxygen species (ROS) produced by nanoparticles in MCF-7 cell line***

According to Table 3 and the averages obtained from the ROS test, it has been found that the treatment of cells with cisplatin increases the amount of intracellular ROS by 63.20%. Silver-graphene nanocomposites, graphene, and silver nanoparticles at 15 μg/ml increased ROS by 31.80%, 30%, and 28.80%, respectively, which were all statistically significant compared with the cisplatin group (*P*<0.0001). With an increasing concentration of the three therapeutic substances, we see an increase in intracellular ROS production, which inhibits cancer cell growth. At the highest therapeutic concentration of 250 μg/ml, this rate reached 68%, 68.40%, and 70.40%. 

**Figure 3:**
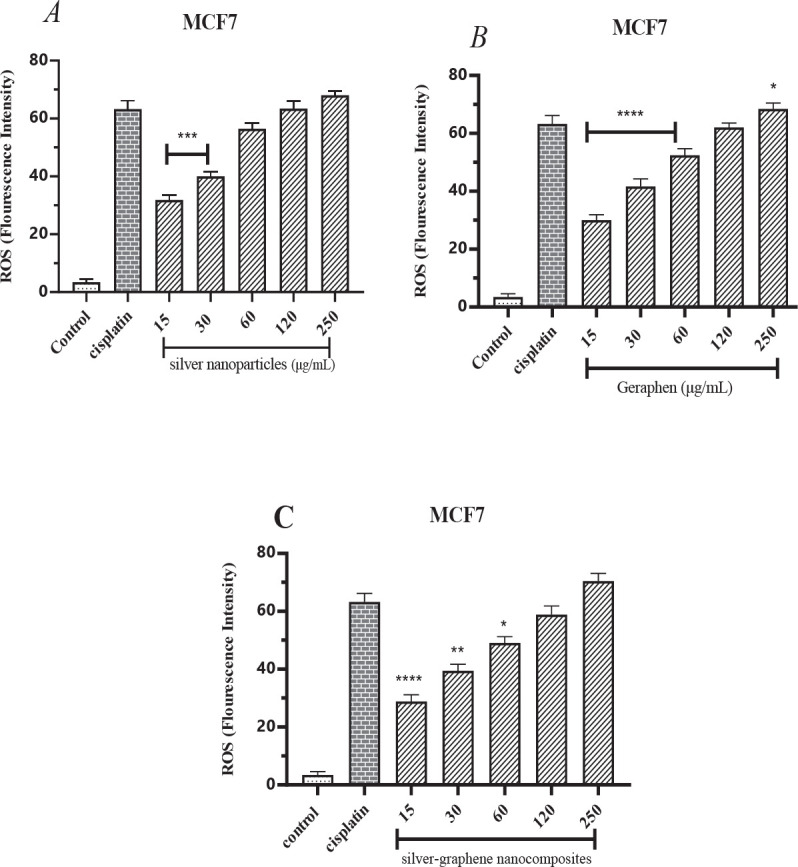
A. The findings gathered from measuring the amount of reactive oxygen species (ROS) caused by silver nanoparticles in the MCF-7 breast cancer cell line using the ELISA device at 420 wavelengths. * (*P*<0.05), ***(*P*<0.001), ****(*P*<0.0001) compared to cisplatin group. B. The findings gathered from measuring the amount of reactive oxygen species (ROS) caused by graphene in the MCF-7 breast cancer cell line using the ELISA device at 420 wavelengths. *(*P*<0.05), ****(*P*<0.0001) compared to cisplatin group. C. The findings gathered from measuring the amount of reactive oxygen species (ROS) caused by silver-graphene nanocomposites in the MCF-7 breast cancer cell line using the ELISA device at 420 wavelengths. * (*P*<0.05), ** (*P*<0.01), ****(*P*<0.0001) compared to cisplatin group

Statistically, in comparison with the cisplatin group, concentrations of 15, 30, 60, and 250 μg/ml silver nanoparticles and graphene (Figure 3A, B) and concentrations of 15, 30, and 60 μg/ml silver-graphene nanocomposites (Figure 3C) were significantly different.

Measuring the quantity of malondialdehyde (MDA) created by nanoparticles in the MCF-7 cell line

**Figure 4:**
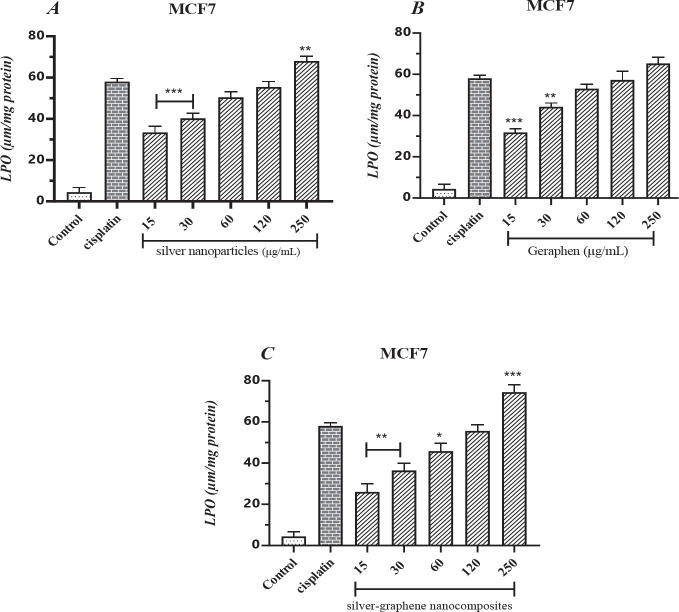
A. The findings gathered from measuring the level of MDA caused by silver nanoparticles in the MCF-7 breast cancer cell line. ** (*P*<0.01), ***(*P*<0.001) compared to cisplatin group. B. The findings gathered from measuring the level of MDA caused by graphene in the MCF-7 breast cancer cell line. ** (*P*<0.01), ***(*P*<0.001) compared to cisplatin group. C. The findings gathered from measuring the level of MDA caused by silver-graphene nanocomposites in the MCF-7 breast cancer cell line. *(*P*<0.05), ** (*P*<0.01), ***(*P*<0.001) compared to cisplatin group

According to Table 4, increased malondialdehyde production, the end product of lipid peroxidation, causes cell toxicity and, eventually, cell death. The amount of MDA produced by cisplatin in MCF cells was 7.58%. In treatment groups with different concentrations of silver nanoparticles, the MDA level increased from 33.40% at a concentration of 15 μg/ml to 68% at 250 μg/ml. It indicates that with increasing concentration, the rate of lipid damage and subsequent cell death has occurred more. Also, in terms of statistical comparison with the cisplatin group, concentrations of 15 and 30 μg/ml (*P*<0.001) and concentrations of 250 μg/ml (*P*<0.01) were significantly different (Figure 4A).

Subsequent treatment of MCF7 cells with graphene at a concentration of 15 micrograms resulted in 31.80% lipid peroxidation, which reached 65.20% at a concentration of 250 micrograms per milliliter. Concentrations of 15 and 30 μg/ml showed significant differences, (*P*<0.001) and (*P*<0.01), respectively (Figure 4B).

In the combination therapy of silver-graphene nanocomposites, the lipid content of lipid damage has increased with increasing concentration, from 26% at the lowest therapeutic concentration to 74.40% at the highest therapeutic concentration. Statistically, concentrations of 15 and 30 (*P*<0.01), 60 (*P*<0.05), and 250 μg/ml (*P*<0.001) were significantly different (Figure 4C).

Calculating the amount of glutathione (GSH) created by nanoparticles in the MCF-7 cell line

Glutathione is a non-enzymatic anti-oxidant. Its role is to reduce the level of oxidants produced in the body. The cell loses its defense power and is destroyed by reducing this factor.

As shown in Table 5, exposure of cancer cells to cisplatin reduces glutathione levels by 80%.

On the other hand, exposure to cells with different concentrations of silver nanoparticles also reduced the glutathione level of MCF7 cells, which increased from 39.60% at 15 μg/ml to 76.40% at 250 μg/ml; Which has shown the effect of silver nanoparticles in reducing glutathione and cancer cell death.

**Figure 5:**
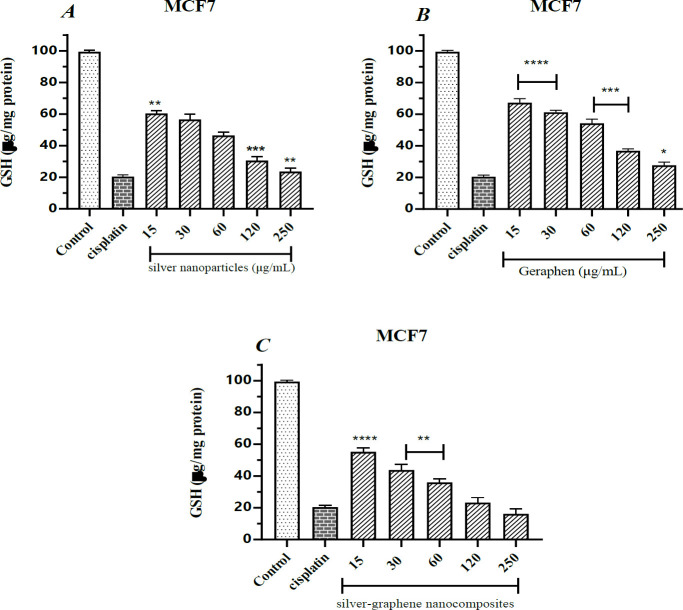
A. The findings gathered from measuring the amount of reactive oxygen species (ROS) caused by silver nanoparticles in the MCF-7 breast cancer cell line using the ELISA device at 420 wavelengths. ** (*P*<0.01), ***(*P*<0.001) compared to cisplatin group.

Also, in statistical analysis and comparison with the cisplatin group, it was found that the concentrations of 15, 120, and 250 μg/ml showed significant differences (*P*<0.01), (*P*<0.001), (*P*<0.01), respectively (Figure 5A).

Graphene-treated cells acted similarly to silver nanoparticles, reducing glutathione by about 73% at a concentration of 250 μg/ml. On the other hand, the statistical comparison showed that all treatment groups had significant differences (Figure 5B).

Combination therapy of silver-graphene nanocomposites significantly reduced glutathione levels and inhibited the growth of cancer cells. At the highest therapeutic concentration, glutathione reserves decreased by about 83.80%, and statistically compared groups of 15, 30, and 60 µg/ml showed a significant difference (*P*<0.0001 and *P*<0.01, respectively) (Figure 5C).

***Calculating the amount of tail moment created by nanoparticles in the MCF-7 cell line***

**Figure 6:**
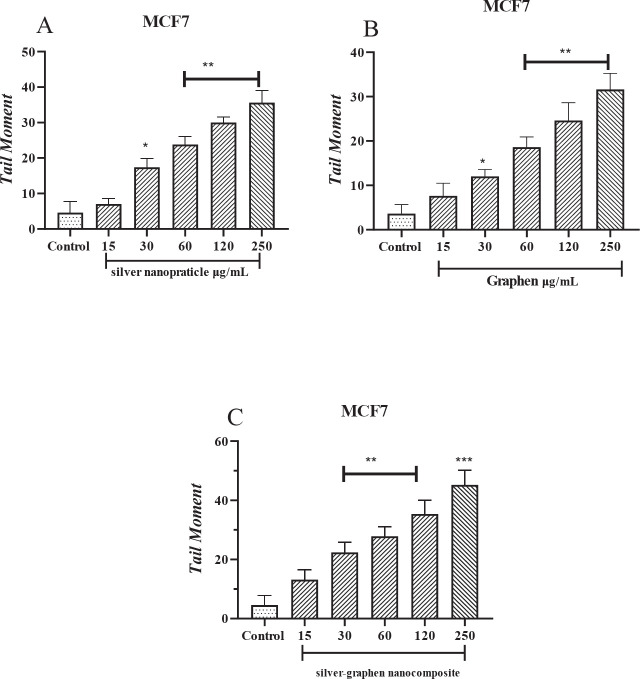
A. Tail moment rate (genetic toxicity) of silver nanoparticle in Mcf-7 cells evaluated by Comet method. * (*P*<0.05), **(*P*<0.01) compared to control group. B. Tail moment rate (genetic toxicity) of graphene in Mcf-7 cells evaluated by Comet method. * (*P*<0.05), **(*P*<0.01) compared to control group. C. Tail moment rate (genetic toxicity) of silver graphene nanoparticles in Mcf-7 cells evaluated by Comet method ** (*P*<0.01), ***(*P*<0.001) compared to cisplatin group

***In th*** e Tail moment graph (Figure 6) compared with the control group, different concentrations of nanoparticles have shown a significant difference (*P*<0.0001).

***Mathematical model***

*Mathematical equation determined for MCF-7 cell line and its treatment with silver nanoparticles, graphene, and silver-graphene nanocomposites*

All three treatments describe a logarithmic nonlinear model. Based on the curve, it is shown that the use of silver-graphene nanocomposite was more effective than silver nanoparticles, and silver nanoparticles were more effective than graphene and had more lethality (Table 6-9).

According to Figure 8, Silver Graphene Nanocomposite> Silver Nanoparticle> Particle has the best effect in inhibiting the growth of cancer cells.

## Discussion

Cancer is a name given to diseases caused by unstoppable tissue reproduction. Therefore, the cells spread to other tissues using the blood flow, tissues, and the lymphatic system without limiting their proliferation. The abnormal cells form gatherings in other body areas (34). Breast cancer is a disease in which abnormal cells grow uncontrollably in different areas of the breast (35). It is one of the malignancies that our country’s women have suffered for the past four decades (36). 

A study on the hydroalcoholic extract of MO found that its mechanism for inhibiting cell growth is due to increased ROS and induction of apoptosis in colorectal cells (15). Another study examined the effect of ethanolic and aqueous extracts. The results showed that the two extracts had an inhibitory effect on the growth of cancer cells (37).

Nowadays, in nanotechnology, using nanoparticles has helped a lot in diagnosing and treating diseases, especially cancer (1, 2). 

This research determined the cell survival rate using the MTT method. The goal was to assess the anticancer effect of silver nanoparticles, graphene, and silver-graphene nanocomposites in breast cancer cells. For this purpose, the ROS, MDA, and intracellular glutathione levels induced by silver nanoparticles, graphene nanocomposites, and silver-graphene in the MCF-7 breast cancer cell line were measured. 

According to the Tables, Figures, and information provided, it was found that cytotoxicity caused by silver nanoparticles inhibited the growth of cancerous cells (positive control). Additionally, this study found that 250 μg/ml of silver nanoparticles caused an increase in the death of cancer cells compared with the cisplatin group. According to IC_50_, silver nanoparticles have a higher mortality effect on cancer cells by increasing the amount of reactive oxygen species and malondialdehyde, and decreasing glutathione reserves. 

Mata *et al. *assessed the anti-oxidant and anticancer effects of silver nanoparticles on human colon cancer cells (COLO207) and Madin-Darby Canine Kidney (MDCK) cells in 2015. Their results showed that silver nanoparticles have a concentration-dependent inhibitory effect on free radicals. They can also cause morphological changes such as chromatin density, loss of cell membrane potential, and stoppage of the G1/S cell cycle. These alterations ultimately lead to the induction of apoptosis in cancer cells. Also, a high level of reactive oxygen species significantly affects DNA fragmentation and can eventually lead to apoptosis (38).

Silver nanoparticles cause toxicity in MCF-7 cells in a way that by increasing the concentration of nanoparticles, the amount of cell toxicity increases, showing that the effects of this combination are concentration-dependent (39). Silver nanoparticles cause cell toxicity and have dose-dependent stimulation in H1299 cells, and silver nanoparticles significantly impede the growth of the H1299 tumor (40). Karam Sichani *et al., *in a 2013 study, revealed that in the group treated with silver nanoparticles, there was a significant increase in malondialdehyde concentration and a significant decrease in catalase and glutathione peroxidase enzyme activity and these changes were dose-dependent. Silver nanoparticles cause lipid peroxidation and malondialdehyde production by creating free radicals (41).

Silver nanoparticles can disrupt the communication network by stopping the transfer of cell signals (22, 23). Silver nanoparticles can damage DNA, increase the production and expression of caspase 3, and induce cellular apoptosis. Hence, they mediate cellular apoptosis signals to other cells, which is vital in cancer treatment (24).

Studies showed that the contact of silver with the SH group on the cell membrane causes changes in cell morphology and plasma cell membrane permeability. Furthermore, silver nanoparticles can cause cell mortality by affecting the cellular respiration chain. Therefore, they can halt cells (42). 

Most of the inhibitory effects of silver nanoparticles may be on the cell’s mitochondrial oxidative phosphorylation chain. Since the mitochondrial respiratory chain’s activity is higher compared with normal cells, silver nanoparticles can be used as a viable method to treat cancer (43). Silver nanoparticles induce the release of cytochrome C into cytosol by increasing ROS. It increases the expression of the protein caspase and induces apoptosis (23).

AgNPs significantly decreased HePG-2 cell proliferation by inducing apoptosis via caspase-3 activation and PARP cleavage. AgNPs, in a dose-dependent manner, considerably increased the apoptotic cell population (sub-G1). HePG-2 cell apoptosis was triggered by the overproduction of ROS, DNA damage-mediated p53 phosphorylation, and affecting MAPKs and AKT signaling (44).

Comparing these results to the present shows that silver nanoparticles can stop the proliferation of cancer cells. The dose-dependent production of reactive oxygen species and malondialdehyde increases, and glutathione production decreases; these alterations cause toxicity and increase cell mortality in breast cancer cells from the MCF-7 line. 

The inhibitory effects of graphene, through its ability to cause cell toxicity, differed significantly from the cisplatin group. However, 250 μg/ml of graphene increases cell mortality and the number of dead cancer cells. Also, this study demonstrated that the percentage of live cells in groups exposed to 15, 30, 60, 120, and 250 μg/ml graphene was higher than in the cisplatin group. 

Zhang *et al., *in a 2010 reading, showed that graphene and single-walled carbon nanotubes induce cellular toxicity, and these effects are dose and shape-dependent. Graphene has higher metabolic activity in lower concentrations than single-walled carbon nanotubes, whereas, in higher concentrations, it is the opposite. Lactate dehydrogenase levels were significantly higher in single-walled carbon nanotube examples compared with graphene. Additionally, ROS were formed in a dose and time-dependent manner after being exposed to graphene, showing an oxidative stress mechanism. Also, after being exposed to graphene, caspase 3 is activated in a time-dependent manner which shows the ability to induce apoptosis in which shape plays a crucial part (45).

An integrated nanosafety study was reported by Medeiros *et al*., which included synthesizing and characterization of graphene oxide-silver nanoparticle hybrid materials (GO-AgNPs) and assessing their nanoecotoxicity in zebrafish embryos. The results of this study have reported the effects of nanoecotoxicity of GO-AgNPs in a dose-dependent manner in zebrafish embryos. Also, silver nanoparticles cause toxicity of the GO-AgNPs nanohybrid. Besides, the chorion embryo membrane critically impacts the toxicity of GO-AgNPs, and NOM is not adequate to prevent the agglomeration of GO-AgNPs in the exposure medium and mitigates the toxicity of GO-AgNPs (46).

Graphene can induce cell toxicity by reducing mitochondrial pressure potential and increasing reactive oxygen species. Afterward, activating caspase 3 and effective proteins in the lower parts of the chain-like PARP ultimately starts mitochondrial apoptosis. Therefore, by increasing the amount of ROS, graphene activates the signaling pathway for mitogen-activating protein kinase, TGF-β, and the caspase 3 pathway through waterfall apoptosis dependent on mitochondria and ultimately induces apoptosis(47). Graphene oxide reduces the life span of cervical cancer cells, mice fibroblast cells, and breast cancer cells from the MCF-7 and SKBR3 cell lines in which all of the said effects were concentration and time-dependent(48). Another study showed that graphene causes evident cell toxicity in a dose-dependent manner which causes the reduction of cell life, increase of ROS, and the release of lactate dehydrogenase in breast cancer cells (49).

After being exposed to graphene materials, the activity of SOD and GPx enzymes reduced in a concentration and time-dependent manner. Oxidative stress is the key reason for apoptosis and DNA damage in cells exposed to graphene materials (45). Reactive oxygen species are produced in response to different stimulants, such as graphene. Due to the surface properties of graphene, a reductive reaction occurs, creating reactive oxygen species (50).

Graphene exposure led to a remarkable time- and concentration-dependent rise in ROS. Based on apoptosis rate, Nrf-2 decrease, ROS, and cytomorphological changes, graphene has a substantial cytotoxic effect against osteosarcoma. Graphene-related cytotoxicity may be enhanced by targeting the IGF1 and IGFBP3 signaling pathways and prolonging the survival of patients with this malignancy (51).

Graphene suppresses the proliferation of MCF-7 cells by activating mitochondrial and NF-κB signaling pathways and leads to programmed death. According to the studies’ results, graphene can be used as a potent synergistic agent in breast cancer treatment (52).

Comparing these results to the present shows that graphene can inhibit cancer cells, and increasing the concentration also increases the inhibitory effects; the dose-dependent production of reactive oxygen species and malondialdehyde increases, and glutathione production decreases. It, in turn, causes toxicity and, ultimately, cell death in breast cancer cells.

The inhibitory effects of silver-graphene nanocomposite through its ability to cause cell toxicity significantly differed from the cisplatin group. At concentrations of 120 and 250 μg/ml, silver-graphene nanocomposite cell mortality and the number of dead cancer cells increased compared with the cisplatin group. Also, this study demonstrated that the percentage of live cells in groups exposed to 120 and 250 μg/ml silver-graphene nanocomposite was lower than in the cisplatin group. 

rGo-Ag nanocomposite significantly impairs the life of cancer cells in ovarian cancer A27080. It increases the release of lactate dehydrogenase, production of reactive oxygen species, activity of caspase 3, and fragmentation of DNA compared with other tested nanomaterials such as graphene oxide, rGo, and AgNP (53).

Another study shows that rGo-AgNP has significant toxic effects against A549 cells with an IC_50_ of 30 μg/ml (54). Graphene oxide nanocomposite coated with 8-hydroxyquinoline significantly increases cell death in colon cancer and breast cancer cells, especially from the MCF-7 cell line, compared with breast cancer cells from a normal line. The toxic effects of nanocomposite are dose-dependent and have a very minimal toxic effect on breast cancer cells from a normal line. One of the main mechanisms of this toxic impact is inducing apoptosis in the cells in a way that starts molecular and cellular apoptosis in cancer cell lines. In contrast, it barely had the same effect on normal cells. The increase in the expression of apoptosis genes such as Bax, P21, and P53 and the reduction in the expression of anti-apoptosis genes such as BCL2 occurs when cancer cells from the MCF-7 line are exposed to nanocomposites. This change in expression levels is not seen in normal breast cells (55).

According to a research, silver nanowire/reduced graphene oxide(AgNWs/rGO) composites affect the structure and esterase-like activity of Human Serum Albumin (HSA) and Human Endometrial Stem Cells (hEnSCs) (56).

Silver-graphene nanocomposites (GO-AgNPs) induced significant cytotoxicity by ROS production, loss of cell viability, increased expression of pro-apoptotic and decreasing expression of anti-apoptotic genes and DNMT3A, increased leakage of LDH, and level of MDA, GO-AgNPs incited DNA hypomethylation in CFFCs (57).

Comparing these results demonstrates that silver-graphene nanocomposites can impede cancer cells, and this inhibitory effect increases by raising the concentration. Reactive oxygen species and malondialdehyde are produced dose-dependently, and the production of glutathione reduces, which in turn causes toxicity and ultimately leads to apoptosis in breast cancer cells.

The results show that using silver nanoparticles and graphene alone or in combination can be a new solution in cancer treatment. As seen in the results, treatment of breast cancer cells with silver or graphene nanoparticles and combination treatment with these two substances against cisplatin have sound effects, and by affecting oxidative stress factors such as increased ROS and subsequent increase in lipid membrane damage, inhibit cell growth and proliferation.

**Table 1:** Particle size, zeta potential, and size distribution of particles measured by Zetasizer

Size	PDI	Zeta potential
110±1.45 nm	0.28±0.02	45±2.1 mV

**Table 2:** Mean and standard deviation of control groups (negative and positive) and different concentrations of silver nanoparticles, graphene and silver-graphene nanocomposites on the MCF-7 breast cancer cell line using the MTT test (3-(4,5-dimethylthiazol-2-yl)-2,5-diphenyltetrazolium bromide)

		**Control**	**cisplatin**	**15**	**30**	**60**	**120**	**250**
silver nanoparticles	Mean	100.0	47.80	61.40	54.20	46.60	30.60	22.80
	Std. Deviation	0.000	2.588	1.673	1.924	3.647	2.408	2.387
graphene	Mean	100.0	47.80	74.40	62.60	53.00	41.20	31.60
	Std. Deviation	0.000	2.588	2.302	2.074	2.236	1.304	1.673
silver-graphene nanocomposites	Mean	*100.0*	*47.80*	*54.40*	*44.20*	*34.80*	*21.20*	*13.60*
	Std. Deviation	*0.000*	*2.588*	*4.037*	*3.271*	*4.438*	*2.588*	*2.302*

**Table 3:** The mean and standard deviation of reactive oxygen species (ROS) in different concentrations of silver nanoparticles, graphene and silver-graphene nanocomposites on the MCF-7 breast cancer cell

		**Control**	**cisplatin**	**15**	**30**	**60**	**120**	**250**
silver nanoparticles	Mean	3.400	63.20	31.80	40.00	56.40	63.40	68.00
	Std. Deviation	1.140	2.950	1.789	1.581	2.074	2.702	1.581
graphene	Mean	3.400	63.20	30.00	41.60	52.40	62.00	68.40
	Std. Deviation	1.140	2.950	1.871	2.702	2.302	1.581	2.074
silver-graphene nanocomposites	Mean	3.400	63.20	28.80	39.40	49.00	58.80	70.40
	Std. Deviation	1.140	2.950	2.387	2.302	2.236	3.033	2.702

**Table 4:** The mean and standard deviation of Malondialdehyde (MDA)in different concentrations of silver nanoparticles, graphene and silver-graphene nanocomposites on the MCF-7 breast cancer cell

		**Control**	**cisplatin**	**15**	**30**	**60**	**120**	**250**
silver nanoparticles	Mean	4.400	58.00	33.40	40.20	50.40	55.40	68.00
	Std. Deviation	2.302	1.581	2.966	2.588	2.702	2.702	2.345
graphene	Mean	4.400	58.00	31.80	44.20	53.00	57.20	65.20
	Std. Deviation	2.302	1.581	1.789	1.924	2.236	4.207	3.114
silver-graphene nanocomposites	Mean	4.400	58.00	26.00	36.40	45.80	55.60	74.40
	Std. Deviation	2.302	1.581	4.062	3.578	3.834	3.050	3.647

Figure 5B. The findings gathered from measuring the amount of reactive oxygen species (ROS) caused by graphene in the MCF-7 breast cancer cell line using the ELISA device at 420 wavelengths. ** (*P*<0.01), ***(*P*<0.001) compared to cisplatin group.

Figure 5C. The findings gathered from measuring the amount of reactive oxygen species (ROS) caused by silver-graphene nanocomposites in the MCF-7 breast cancer cell line using the ELISA device at 420 wavelengths. ** (*P*<0.01), ***(*P*<0.001) compared to cisplatin group

**Table 5:** the mean and standard deviation of glutathione (GSH) in different concentrations of silver nanoparticles, graphene and silver-graphene nanocomposites on the MCF-7 breast cancer cell

		**Control**	**cisplatin**	**15**	**30**	**60**	**120**	**250**
silver nanoparticles	Mean	99.40	20.40	60.40	56.60	46.40	30.60	23.60
	Std. Deviation	0.8944	1.140	1.817	3.435	2.074	2.408	2.302
graphene	Mean	99.40	20.40	67.20	61.20	54.20	36.80	27.60
	Std. Deviation	0.8944	1.140	2.588	1.304	2.683	1.304	2.074
silver-graphene nanocomposites	Mean	99.40	20.40	55.20	43.80	36.00	23.20	16.20
	Std. Deviation	0.8944	1.140	2.508	3.421	2.236	3.271	3.114

**Table 6:** Summary of statistical analysis output for regression models related to MCF7 cell line treated with silver nanoparticles

	Parm	Value	Std Error	t-value	99% Confidence Limits	P>|t|
Silver* nanoparticles* MCF-7	a	102.0831030	2.738093030	37.28255462	94.39635689	109.7698490
	b	-14.3724495	0.648728137	-22.1548114	-16.1936471	-12.5512519
	**r2 Coef Det**	**DF Adj r2**	**Fit Std Err**	**F-value**	**F-value**	
	0.9552386076	0.9511693901	3.2172749219	490.83566845	0.9552386076	
	**Source**	**Sum of Squares**	**DF**	**Mean Square**	**F Statistic**	**P>F**
	**Regr**	5080.5703	1	5080.5703	490.836	0.00000
	**Error**	238.06973	23	10.350858		
	**Total**	5318.64	24			

**Table 7:** Summary of statistical analysis output for regression models related to MCF7 cell line treated with graphene

	Parm		Value	Std Error	t-value	99% Confidence Limits	P>|t|
**MCF-7**	a		115.1290392	1.656888273	69.48509510	110.4775978	119.7804807
	b		-15.2514083	0.392561549	-38.8509989	-16.3534603	-14.1493563
	**r2 Coef Det**		**DF Adj r2**	**Fit Std Err**	**F-value**	**r2 Coef Det**	
	0.9849908651		0.9836263983	1.9468531682	1509.4001121	0.9849908651	
	**Source**	**Sum of Squares**		**DF**	**Mean Square**	**F Statistic**	**P>F**
	**Regr**		5720.9845	1	5720.9845	1509.4	0.00000
	**Error**		87.175457	23	3.7902373		
	**Total**		5808.16	24			

**Figure 7:**
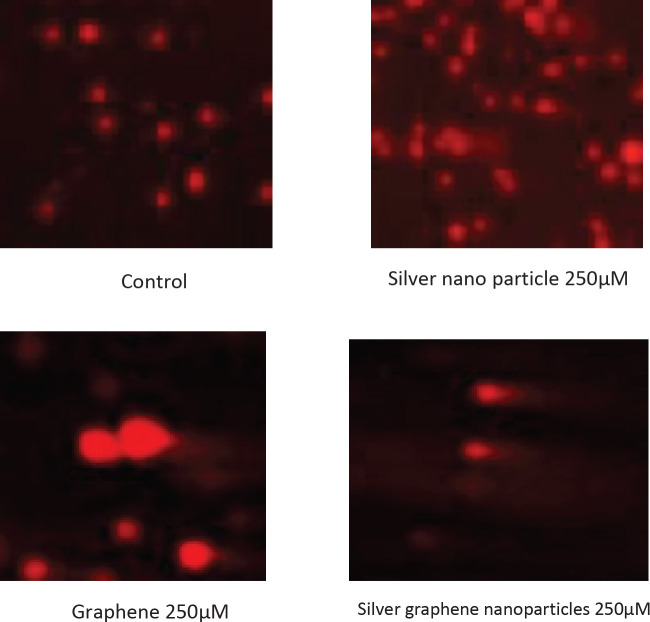
Photomicrographs of MCF-7 cells were treated to silver nanoparticles, graphene and silver graphene nanoparticles

**Table 8:** Summary of statistical analysis output for regression models related to MCF7 cell line treated with silver-graphene nanocomposites

	Parm		Value	Std Error	t-value	99% Confidence Limits	P>|t|
*silver-graphene nanocomposites * **MCF-7**	a		94.79030541	2.922824665	32.43106114	86.58495565	102.9956552
	b		-14.9055873	0.692496047	-21.5244366	-16.8496561	-12.9615185
	**r2 Coef Det**		**DF Adj r2**	**Fit Std Err**	**F-value**	**r2 Coef Det**	
	0.9527042257		0.9484046098	3.4343356463	463.30137305	0.9527042257	
	**Source**	**Sum of Squares**		**DF**	**Mean Square**	**F Statistic**	**P>F**
	**Regr**		5464.4828	1	5464.4828	463.301	0.00000
	**Error**		271.27721	23	11.794661		
	**Total**		5735.76	24			

**Figure 8:**
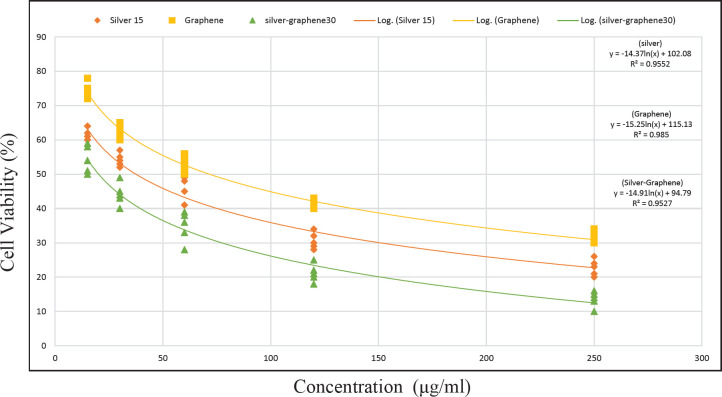
Diagram of treatment MCF-7 cell line by silver nanoparticles, graphene and silver-graphene nanocomposite. y axis is about cell viability and X axis is about concentration of substance. The square symbol represents the graphene curve, the rhombus symbol represents the silver curve and the triangle symbol represents the silver graphene. And according to the shape, Silver Graphene Nanocomposite> Silver Nanoparticle> Particle has the best effect in inhibiting the growth of cancer cells

(silver) y = -14.37ln(x) + 102.08 - R² = 0.9552

(Graphene) y = -15.25ln(x) + 115.13 - R² = 0.985

(Silver-Graphene) y = -14.91ln(x) + 94.79 - R² = 0.9527

**Table 9:** Mathematical Model of MCF7 cell line treated with silver nanoparticles, Graphene and silver-graphene nanocomposites

MCF-7 cell line	
Silver nanoparticles	y=a+blnx
Graphene	y=a+blnx
silver-graphene nanocomposites	y=a+blnx

## Conclusion

Treatment of MCF-7 cells with silver or graphene nanoparticles and combination treatment with these two substances against cisplatin have sound effects, and by affecting oxidative stress factors such as increased ROS and subsequent increase in lipid membrane damage, inhibit cell growth and proliferation. According to the mathematical model, silver graphene nanocomposite> silver nanoparticles> graphene has the best effect in inhibiting the growth of cancer cells.

Characterization techniques were not enough; FTIR analyses of extract, Ag- NPs, and silver-graphene nanocomposites could be performed in future studies to confirm functional groups and, more precisely, the chemical structure, which will guide researchers to discover the molecular mechanism of the mentioned anticancer effects. Also, we could not evaluate the anticancer effects of* M. officinalis* in other cell lines (HepG2 liver cancer, A549 liver cancer, etc.). It is possible to measure *M. officinalis*’s anticancer effect on a larger scale by examining different cell lines and signaling pathways associated with apoptosis.

## Authors’ Contributions

MS and MG Contributed to conception, study design, and management. BK Contributed to the mathematical model. FM Contributed to the analysis of data, writing the article, and drafting the manuscript. FM and PM Contributed to the preparation of the cell line, stress oxidative assay, and editing the manuscript. NGB Contributed to the comet assay and revised the manuscript. All authors read and approved the final manuscript. 

## Availability of Data and Materials

The authors confirm that the data supporting the findings of this study are available.

## Funding

This study was supported by a grant from the Research Council of Mazandaran University of Medical Sciences (grant no. 2652534).

## Conflicts of Interest

All authors declare that they have no conflicts of interest.
